# Performance evaluation of DF-based cooperative DCSK for next-generation wireless networks

**DOI:** 10.1038/s41598-026-50539-6

**Published:** 2026-05-22

**Authors:** Maiss M. Al-Khasawneh, Mamoun F. Al-Mistarihi, Moawiah Alhulayil, Mohammed M. Alammar

**Affiliations:** 1 Department of Electrical Engineering, Jordan University of Science and Technology, Irbid, 22110 Jordan; 2 Department of Electrical Engineering, Applied Science Private University (ASU), Amman, 11937 Jordan; 3 Department of Electrical Engineering, King Khalid University, 61421 Abha, Saudi Arabia

**Keywords:** Engineering, Mathematics and computing

## Abstract

Differential chaos shift keying (DCSK) is a non-coherent modulation technique that has attracted attention for communication over fading channels. This paper investigates the performance of a cooperative DCSK system employing decode-and-forward (DF) relaying over Rayleigh and Nakagami-*m* fading environments. Analytical expressions are developed for outage probability and average-capacity evaluation, while the bit error performance is examined through an analytical treatment supported by Monte Carlo simulation. The analysis shows that the Nakagami-*m* fading parameter has a clear impact on system performance, where less severe fading leads to improved reliability. The results also indicate that the relay-destination link plays an important role in the cooperative gain achieved by the considered system. The contribution of the paper is presented within the scope of the adopted two-user DF cooperative DCSK model and the corresponding analytical assumptions. Therefore, the reported findings should be interpreted as an analytical and numerical characterization of the studied framework under the specified channel conditions.

## Introduction

Wireless communication performance is heavily affected by signal distortions like multipath fading and intersymbol interference (ISI), which are common in real-world propagation scenarios^1,2^. Multipath fading arises when transmitted signals arrive at the receiver through multiple paths of varying delays, amplitudes, and phases, resulting in destructive or constructive interference that degrades signal quality^3–7^.

To mitigate these impairments, diversity techniques have become an essential design element in modern wireless systems. Among them, multiple-input multiple-output (MIMO) systems offer improved link reliability and capacity by leveraging spatial diversity using multiple antennas at both ends of the communication link^8–10^. However, in scenarios constrained by hardware size, cost, and power, for applications involving wireless sensor networks (WSNs) and Internet of Things (IoT)-enabled systems, MIMO implementations may be infeasible.

To address these limitations, cooperative communication has emerged as a scalable alternative. In cooperative diversity, single-antenna nodes collaborate by relaying each other’s messages, effectively creating a virtual MIMO system^11–13^. This approach improves reliability and performance under fading conditions without requiring additional antenna hardware. Common cooperative strategies such as amplify-and-forward (AF) and decode-and-forward (DF) are widely adopted to support these operations^14–18^.

Recent research has explored the integration of chaos-based modulation techniques, especially differential chaos shift keying (DCSK), within cooperative systems. Chaotic signals exhibit broadband spectra, are non-periodic, and are highly sensitive to initial conditions, which has motivated their use in interference-resilient communication studies^19–22^. DCSK, in particular, is valued for its simplicity and robustness since it operates without the need for channel estimation or synchronization with the carrier signal^23,24^.

Although cooperative DCSK systems have been investigated to some extent, their behavior under practical multipath fading environments warrants further analysis. Fading, influenced by environmental scattering, shadowing, and mobility, introduces amplitude and phase fluctuations in the received signal. While coherent modulation schemes suffer from phase tracking errors, non-coherent schemes like DCSK primarily contend with envelope fading^25–28^. On the other hand, fading channels are further categorized based on time and frequency selectivity. Slow and fast fading are characterized by the channel’s coherence time and doppler spread. In contrast, the nature of the channel, flat or frequency-selective, is governed by its coherence bandwidth and delay spread characteristics^29–33^. Rayleigh fading is commonly used to model scenarios where the line-of-sight (LOS) signal is either weak or completely obstructed, while Nakagami-$m$ fading offers flexibility through a shape parameter $m$ and includes Rayleigh fading as a limiting scenario when the fading parameter is equal to one^34–41^.

The combination of cooperative communication and DCSK modulation has been examined in several prior studies under different channel assumptions and system configurations. The discussion below focuses on the studies most directly related to the considered cooperative DCSK framework, particularly under Rayleigh and Nakagami-*m* fading channels.

Cooperative DCSK systems have been examined under different channel settings and cooperation structures. Proposed^42^ a two-user DCSK cooperative communication system with decode-and-forward relaying and analyzed its bit error performance over multipath Rayleigh fading channels. Extended^43^ the cooperative DCSK framework to a multiple-access setting over Nakagami-*m* fading channels and provided BEP and throughput analysis. Investigated^44^ the bit error probability of a two-user DCSK cooperative communication system over Nakagami-*m* fading channels and examined the influence of fading severity on the resulting performance. In a broader extension,^45^ studied DCSK MIMO relay cooperative diversity under Nakagami-*m* fading and generalized Gaussian noise, with analytical error-rate results for multiple relaying protocols. Since these studies differ in system structure, channel assumptions, and analytical emphasis, a comparative summary is provided in Table 1.

To clarify the position of the present study relative to the most relevant prior contributions, Table 1 summarizes the main differences in terms of system setting, fading model, and analyzed performance metrics, with particular attention to works closely related in topic and analytical scope.

**Table 1:** Comparison of the present study with selected prior works on cooperative DCSK systems.

Refs.	Primary focus	System/scenario	Fading model	Analyzed metrics	Main distinction relative to this work
^42^	Two-user cooperative DCSK performance	Two-user cooperative DCSK with DF relaying	Multipath Rayleigh	BEP	Focused on BEP analysis of a two-user cooperative DCSK architecture over multipath Rayleigh fading, without addressing outage probability or average-capacity behavior
^43^	Multiple-access cooperative DCSK analysis	Multiple-access DCSK-CC system	Nakagami-*m*	BEP and throughput	Considered a multiple-access cooperative framework and emphasized BEP and throughput, rather than the fixed two-user setting and broader combined metric evaluation considered here
^44^	BEP evaluation for cooperative DCSK	Two-user cooperative DCSK system	Nakagami-*m*	BEP	Focused specifically on BEP under Nakagami-*m* fading, without including outage probability or average-capacity analysis
^45^	MIMO relay cooperative diversity in DCSK	DCSK MIMO relay cooperative diversity	Nakagami-*m* and generalized Gaussian noise	BEP	Examined a broader MIMO relay diversity setting with multiple relaying protocols, which differs from the fixed two-user DF cooperative DCSK framework considered in this paper
This work	Two-user cooperative DCSK performance study	Fixed two-user DF cooperative DCSK	Rayleigh and Nakagami-*m*	BEP, outage probability, and average- capacity behavior	Provides a consolidated analytical and numerical study of the considered fixed two- user DF cooperative DCSK model under the adopted assumptions

This study investigates the performance of a cooperative two-user DCSK system employing decode-and-forward (DF) relaying over Rayleigh and Nakagami-*m* fading channels. Rather than claiming a fundamentally new cooperative architecture, the contribution of this work is to provide a unified analytical and numerical treatment of three commonly used performance measures within the considered framework, namely BEP, outage probability, and average-capacity behavior, under the assumptions adopted in the manuscript. Therefore, the presented results should be interpreted as an analytical study of the considered DF-based cooperative DCSK model under the specified channel assumptions, rather than as a universal characterization of all cooperative DCSK systems. For completeness, the basic DCSK transceiver structure used as the basis for the subsequent cooperative system model is briefly recalled next.

The analysis presented in this paper is subject to the modeling assumptions adopted for analytical tractability. In particular, the results are derived for the considered two-user DF cooperative DCSK framework under Rayleigh and Nakagami-*m* fading conditions and based on the corresponding signal and SNR formulations used throughout the manuscript. Moreover, the present study does not address implementation complexity, protocol overhead, synchronization impairments, hardware constraints, secrecy aspects, or detailed energy-consumption analysis. Therefore, the conclusions of this work should be interpreted within the limits of the adopted analytical model and should not be generalized beyond the considered framework without further investigation.

**Fig. 1:**
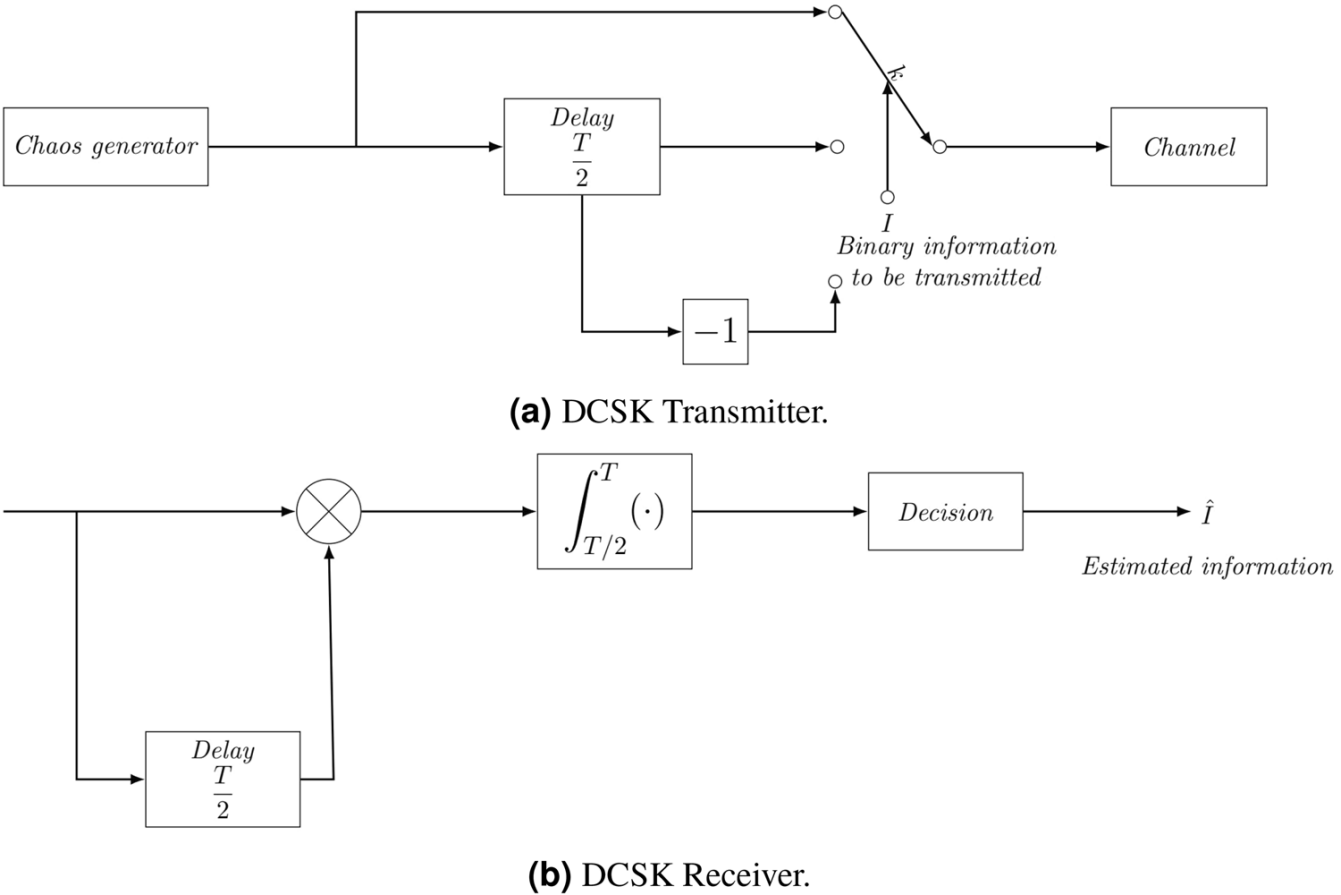
Block schematic of a DCSK-based transceiver.

Figure 1 illustrates a typical DCSK transceiver, which consists of a transmitter and receiver pair. DCSK is a non-coherent modulation scheme that transmits one chaotic reference and one data-modulated signal in each bit duration. The chaotic sequence serves both as a carrier and reference, eliminating the need for synchronization.

The transmitted signal is represented using orthogonal basis functions $g_1(t)$ and $g_2(t)$ as follows

1 $$\begin{aligned} s(t) = s_{m1}g_1(t) + s_{m2}g_2(t) \end{aligned}$$

with

2 $$\begin{aligned} g_1(t)&= {\left\{ \begin{array}{ll} c(t), & 0 \le t< T/2 \\ c(t - T/2), & T/2 \le t < T \end{array}\right. } \end{aligned}$$

3 $$\begin{aligned} g_2(t)&= {\left\{ \begin{array}{ll} c(t), & 0 \le t< T/2 \\ -c(t - T/2), & T/2 \le t < T \end{array}\right. } \end{aligned}$$

where $c(t)$ is a chaotic sequence and $T$ is the bit period. At the receiver, a suboptimal decision variable is computed using an autocorrelation detector as follows

4 $$\begin{aligned} z = \int _{T/2}^{T} r(t) r(t - T/2) \, dt \end{aligned}$$

where $r(t)$ is the received signal. A positive $z$ denotes bit 1, while a negative $z$ denotes bit 0.

The main contributions of this study are summarized as follows:

- A performance study of a two-user cooperative DCSK system over Rayleigh and Nakagami-$m$ fading channels within a fixed decode-and-forward relaying framework.
- Development of analytical results for outage probability and average-capacity evaluation, together with an analytical treatment of BEP under the adopted model assumptions.
- Verification of the analytical results through Monte Carlo simulation and examination of the effect of fading severity on the considered system behavior.

The structure of the paper is as follows: Section “System model” introduces the system model. Section “Performance analysis” presents the analytical evaluation of performance metrics. Section “Results and discussion” discusses the simulation outcomes and comparative analysis. Lastly, Section “Conclusion and future work” summarizes the key findings and outlines future research directions.

## System model

A two-user cooperative framework utilizing differential chaos shift keying (DCSK) is the focus of this investigation, where cooperation is achieved using a DF relay protocol. Each user not only transmits its own signal but also serves as a relay for the other, thus forming a virtual spatial diversity system without the need for multiple antennas. Cooperation occurs in two separate stages, namely the broadcast phase and the cooperation phase, both conducted over frequency-selective channels modeled using Rayleigh or Nakagami-*m* statistics. To maintain orthogonality between concurrent transmissions, Walsh codes are employed at the signaling level throughout the communication process.

In general, there are two cooperation schemes for communication. In the conventional cooperation protocol, the time is partitioned into odd and even periods. In the broadcast phase (odd time slots), each user transmits its own data, which is received by both the base station and the other user. During the cooperative phase (even time slots), each user forwards the decoded information received from the other to the base station. This configuration helps to improve the overall signal-to-noise ratio (SNR) and achieve diversity gains. Alternatively, a space-time cooperation protocol may be used where both the user’s signal and the estimated signal from its partner are transmitted simultaneously using space-time block coding. We consider here a two-user cooperative DCSK system in which both users transmit their own signals and act as decode-and-forward (DF) relays for one another.

**Fig. 2:**
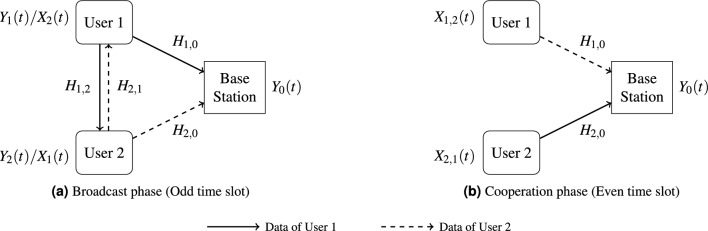
System model of two-user cooperative DCSK communication.

The communication process operates over two interleaved time intervals: the *odd period* (broadcast phase) and the *even period* (cooperation phase). The detailed transmission process is depicted in Fig.  2.

The following assumptions are adopted throughout the analysis. The links $U_1\!\rightarrow \!\textrm{BS}$, $U_2\!\rightarrow \!\textrm{BS}$, and the inter-user link are assumed statistically independent. Within each link, the multipath components are taken to be independent, while the per-link fading severity is described by the link parameters $m_1$, $m_2$, and $m_3$, respectively. Unless otherwise stated, equal transmit energy is assumed for the two users, and the fading coefficients are considered quasi-static over one DCSK symbol interval. Perfect timing alignment at the decision stage is also assumed so that the analysis can focus on fading-induced performance trends. In addition, the choice $L=3$ is adopted as a tractable representative multipath setting that is consistent with the three-ray DCSK modeling used in several earlier cooperative DCSK studies and allows a compact analytical treatment.

Let $X_i(t)$, for $i \in \{1,2\}$, denote the transmitted signal from user $i$, and let $Z_i(t)$ represent additive white Gaussian noise (AWGN) with zero mean and two-sided power spectral density $N_i/2$. The base station is denoted by index 0.

The received signals at the base station, user 1, and user 2 during the odd period are represented by $Y_0^{\text {odd}}(t)$, $Y_1(t)$, and $Y_2(t)$, respectively, and are given by

5 $$\begin{aligned} \begin{aligned} Y_1(t)&= H_{2,1}(t) \otimes X_2(t) + Z_1(t), \\ Y_2(t)&= H_{1,2}(t) \otimes X_1(t) + Z_2(t), \\ Y_0^{\text {odd}}(t)&= H_{1,0}(t) \otimes X_1(t) + H_{2,0}(t) \otimes X_2(t) + Z_0^{\text {odd}}(t). \end{aligned} \end{aligned}$$

Each user forwards the other’s decoded data to the base station during the even period. Let $X_{1,2}(t)$ denote the signal relayed by user 1 on behalf of user 2, and $X_{2,1}(t)$ the signal relayed by user 2 on behalf of user 1. In the cooperation phase, the received signal at the base station is given by

6 $$\begin{aligned} Y_0^{\text {even}}(t) = H_{1,0}(t) \otimes X_{1,2}(t) + H_{2,0}(t) \otimes X_{2,1}(t) + Z_0^{\text {even}}(t). \end{aligned}$$

The wireless channels between nodes are modeled as linear time-invariant multipath channels with impulse responses $H_{i,j}(t)$, given by

7 $$\begin{aligned} H_{i,j}(t) = \sum _{l=1}^{L} \alpha _l^{(i,j)} \delta \!\left( t - \tau _l^{(i,j)}\right) \end{aligned}$$

where $L$ indicates the number of channel paths due to multipath propagation, and $\alpha _l^{(i,j)}$ and $\tau _l^{(i,j)}$ represent the gain and delay of the $l$-th path between transmitter $i$ and receiver $j$, respectively.

During the even period, user 1 uses $Y_1(t)$ to decode and reconstruct user 2’s data and transmits it as $X_{1,2}(t)$ to the base station. Similarly, user 2 reconstructs user 1’s data from $Y_2(t)$ and transmits $X_{2,1}(t)$.

This cooperative transmission strategy improves link reliability through spatial diversity. The present analysis assumes ideal timing alignment at the detector and does not explicitly model synchronization errors, implementation losses, or unequal power allocation. These practical aspects are therefore outside the scope of the current manuscript.

## Performance analysis

### Channel PDF derivation

To begin the performance analysis, we introduce the probability density function (PDF) used for the equivalent received SNR at the users and at the base station. For analytical tractability, the number of multipath components is fixed to $L = 3$. The channel gains are assumed independent, but not necessarily identically distributed across the three communication links. In the numerical study, $m_1$, $m_2$, and $m_3$ denote the per-link Nakagami-$m$ parameters of the source–destination, relay–destination, and inter-user links, respectively.

The PDF of each path-level fading coefficient $\alpha _l$ is written as

8 $$\begin{aligned} f(\alpha _l) = \frac{2m_l^{m_l}}{\Gamma (m_l)\bar{\alpha }_l^{m_l}} \alpha _l^{2m_l-1} \exp \left( -\frac{m_l \alpha _l^2}{\bar{\alpha }_l} \right) , \end{aligned}$$

where $\Gamma (\cdot )$ denotes the Gamma function,

9 $$\begin{aligned} \Gamma (z) = \int _0^\infty t^{z-1} e^{-t} dt, \quad z > 0. \end{aligned}$$

The instantaneous SNR of each path is defined as $\gamma _l = \frac{E_b}{N_0}\alpha _l^2$, with average SNR $\bar{\gamma }_l = \frac{E_b}{N_0}\mathbb {E}[\alpha _l^2]$. Accordingly, the path-level SNR PDF follows the Gamma form

10 $$\begin{aligned} f(\gamma _l) = \frac{m_l^{m_l}\gamma _l^{m_l-1}}{\Gamma (m_l)\bar{\gamma }_l^{m_l}} \exp \left( -\frac{m_l\gamma _l}{\bar{\gamma }_l}\right) . \end{aligned}$$

For compactness, the adopted Nakagami-$m$ representation of the equivalent combined SNR is expressed in the generic form

11 $$\begin{aligned} f_{\gamma _b}(\gamma _b)= c\, e^{-b_1\gamma _b}\gamma _b^{a-1}\,{}_1F_1\!\left( b,a,\beta \gamma _b\right) , \end{aligned}$$

where $a$, $b$, $\beta$, and $c$ are effective parameters determined by the link fading severities and the corresponding average SNR values. The same functional form is used for the base-station equivalent SNR after combining the odd- and even-phase contributions, namely

12 $$\begin{aligned} f_{\gamma _b^{\textrm{BS}}}(\gamma _b)= c_{\textrm{BS}}\, e^{-b_1\gamma _b}\gamma _b^{a_{\textrm{BS}}-1}\,{}_1F_1\!\left( b_{\textrm{BS}},a_{\textrm{BS}},\beta _{\textrm{BS}}\gamma _b\right) . \end{aligned}$$

This notation is adopted to keep the subsequent outage and average-capacity derivations compact and to avoid ambiguity between path-level parameters and link-level parameters. “Appendix A” summarizes the corresponding modeling steps.

### Bit error probability (BEP)

This subsection derives an analytical lower-bound treatment for the bit error probability (BEP) of the considered DCSK cooperative system under a DF relay protocol and a two-phase cooperation scheme consisting of the *broadcast phase* and the *cooperation phase*.

During broadcasting, each user’s transmission is detected by the base station and the peer user. Specifically, the signal from User 1 is received at User 2 as

13 $$\begin{aligned} Y_2(t) = H_{1,2}(t) \otimes X_1(t) + Z_2(t) \end{aligned}$$

User 2 then makes a hard decision to estimate $\hat{b}^{(1)}$ of User 1. The BEP is expressed as

14 $$\begin{aligned} P_{e12} = \int _0^\infty P_e(\gamma _b) f_{\gamma _b}(\gamma _b) \, d\gamma _b \end{aligned}$$

where $P_e(\gamma _b)$ is the conditional BEP given instantaneous SNR $\gamma _b$ and $f_{\gamma _b}(\gamma _b)$ is the PDF from Eq. (11).

Given that User 1 transmits a bit ‘1’, the conditional BEP based on the generalized maximum likelihood criterion is expressed as

15 $$\begin{aligned} P_e(\gamma _b) = \Pr (\hat{b} = 0 \mid b = 1, H_{1,2}) = \Pr (E_{1,0} > E_{1,1} \mid H_{1,2}) \end{aligned}$$

Here, $E_{1,0}$ and $E_{1,1}$ denote the decision metrics (or weighted energies) of User 1 corresponding to the transmission hypotheses of bit ‘0’ and bit ‘1’, respectively. When the chaotic sequence is produced via a logistic map, the conditional BEP becomes

16 $$\begin{aligned} P_e(\gamma _b) = \frac{1}{2} \, \text {erfc} \left[ \left( \frac{4}{\gamma _b} + \frac{2\beta }{\gamma _b^2} \right) ^{-\frac{1}{2}} \right] \end{aligned}$$

At the base station, soft decision is applied to the received signals during both phases

17 $$\begin{aligned} Y_o^{\text {odd}}(t)&= H_{1,0} \otimes X_1(t) + H_{2,0} \otimes X_2(t) + Z_o^{\text {odd}}(t) \end{aligned}$$

18 $$\begin{aligned} Y_o^{\text {even}}(t)&= H_{1,0} \otimes X_{1,2}(t) + H_{2,0} \otimes X_{2,1}(t) + Z_o^{\text {even}}(t) \end{aligned}$$

To obtain a lower-bound characterization of the BEP at the base station, we assume that the even-period weighted energy equals the odd-period weighted energy and that the inter-user channel is perfect, i.e., $P_{e12} = 0$. Under these idealized assumptions, the resulting expression represents a lower-bound approximation for the considered system rather than an exact BEP expression.

19 $$\begin{aligned} P_e = \Pr \left( E_{1,0}^{\text {odd}} - E_{1,1}^{\text {odd}} + E_{1,0}^{\text {even}} - E_{1,1}^{\text {even}} > 0 \mid H_{1,0}, H_{2,0} \right) = \text {BEP}(\gamma _b^{\text {odd}}, \gamma _b^{\text {even}}) \end{aligned}$$

The lower-bound BEP at the base station is then obtained numerically by substituting Eqs. (12) and (16) into the integral form of Eq. (14). Please refer to “Appendix B” for more details.

### Outage probability

Outage probability refers to the likelihood that the instantaneous equivalent SNR, $\gamma _b$, drops below a specified threshold $\gamma _{\text {th}}$. It is mathematically represented as

20 $$\begin{aligned} P_{\text {out}} = \Pr (\gamma _b < \gamma _{\text {th}}) = F_{\gamma _b}(\gamma _{\text {th}}) = \int _0^{\gamma _{\text {th}}} f_{\gamma _b}(\gamma _b) \, d\gamma _b. \end{aligned}$$

In the revised manuscript, the Rayleigh case is treated as the special case of the adopted framework obtained when the corresponding Nakagami-$m$ parameters are set to unity. Accordingly, the Rayleigh reference curves reported in the results section are obtained by numerical evaluation of Eq. (20) under $m_1=m_2=m_3=1$, rather than by invoking an external closed-form expression.

For the general Nakagami-$m$ case, substituting Eqs. (12) into  (20) gives

21 $$\begin{aligned} P_{\text {out}} = \int _0^{\gamma _{\text {th}}} c_{\textrm{BS}} e^{-b_1 \gamma }\gamma ^{a_{\textrm{BS}}-1}\,{}_1F_1\!\left( b_{\textrm{BS}},a_{\textrm{BS}},\beta _{\textrm{BS}}\gamma \right) \, d\gamma . \end{aligned}$$

The corresponding numerical reference curves are obtained by evaluating the above integral directly. This presentation avoids unnecessary symbol proliferation and keeps the Rayleigh and Nakagami-$m$ cases within one consistent notation.

### Average-capacity indicator

To complement the BEP and outage analyses, we consider an average-capacity indicator based on the equivalent received SNR of the studied fading model. This quantity is introduced to compare relative performance trends under different channel conditions. It should be interpreted as an SNR-based information-theoretic reference measure, rather than as the exact achievable spectral efficiency of the full DCSK-CC protocol, since the present analysis does not explicitly account for protocol overhead, reference-signal overhead, or half-duplex time normalization.

For an AWGN channel with bandwidth $\textrm{BW}$ and received SNR $\gamma$, the instantaneous reference capacity is

22 $$\begin{aligned} C_\gamma = \textrm{BW}\log _2(1+\gamma ). \end{aligned}$$

For a fading channel, the corresponding average-capacity indicator is obtained by averaging Eq. (22) over the SNR distribution,

23 $$\begin{aligned} \bar{C}_\gamma = \textrm{BW} \int _0^\infty \log _2(1 + \gamma ) \, p_\gamma (\gamma ) \, d\gamma . \end{aligned}$$

In this work, Eqs. (22) and (23) are used as reference expressions to quantify average-capacity trends associated with the equivalent received SNR. They are not intended to represent a fully normalized spectral-efficiency expression for the complete DCSK-CC transmission protocol. In particular, the present formulation does not explicitly include normalization factors associated with the DCSK reference segment or with the half-duplex broadcast/cooperation time split.

For the Rayleigh case, the numerical reference curves are obtained from Eq. (23) by setting $m_1=m_2=m_3=1$ in the adopted equivalent-SNR model. For the general Nakagami-$m$ case, substitution of Eq. (12) yields

24 $$\begin{aligned} \bar{C} = \textrm{BW} \int _0^\infty \log _2(1+\gamma )\, c_{\textrm{BS}} e^{-b_1\gamma }\gamma ^{a_{\textrm{BS}}-1}\,{}_1F_1\!\left( b_{\textrm{BS}},a_{\textrm{BS}},\beta _{\textrm{BS}}\gamma \right) \, d\gamma . \end{aligned}$$

The resulting curves are obtained through direct numerical evaluation of Eq. (24). “Appendix D” summarizes the adopted evaluation steps.

## Results and discussion

This section provides a performance assessment of the proposed DCSK-cooperative communication (DCSK-CC) system under different channel fading conditions using Monte Carlo simulations in MATLAB. The simulations assess bit error probability (BEP), outage probability, and the average-capacity indicator under Nakagami-*m* and Rayleigh fading scenarios. The key parameters adopted for simulation in this study are listed in Table 2. Each result is compared with the corresponding analytical or numerical reference curves to assess the consistency of the theoretical derivations.

**Table 2:** Simulation parameters.

Parameter	Value/description
Spreading factor (*f*)	32, 64
Number of Monte Carlo iterations	$10^6$
Modulation type	DCSK
SNR range	0–20 dB
Nakagami-*m* parameters ($m_1, m_2, m_3$)	Main discussion focused on 0.5 to 1.5; one legacy stress-test case with a very small shaping parameter is retained in the original BEP figures for completeness
Threshold SNR ($\gamma _{\text {th}}$)	10, 12.5, 15 dB (variable)
Channel types	Rayleigh ($m=1$), Nakagami-*m*

**Fig. 3:**
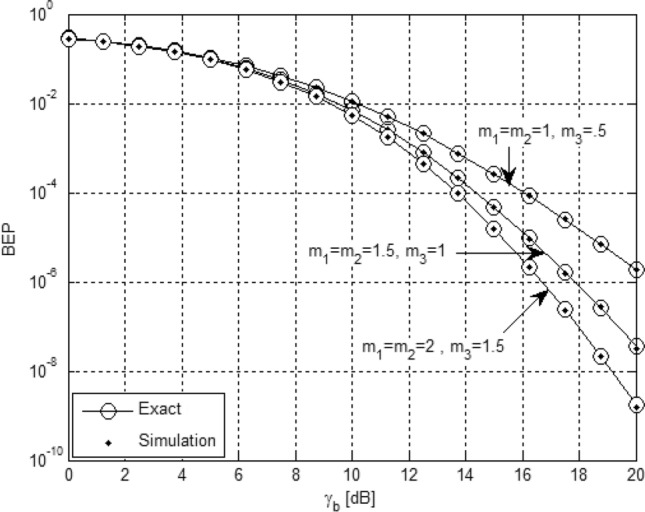
Impact of Nakagami-$m$ severity on the BEP performance of DCSK-CC with spreading factor $f = 32$.

**Fig. 4:**
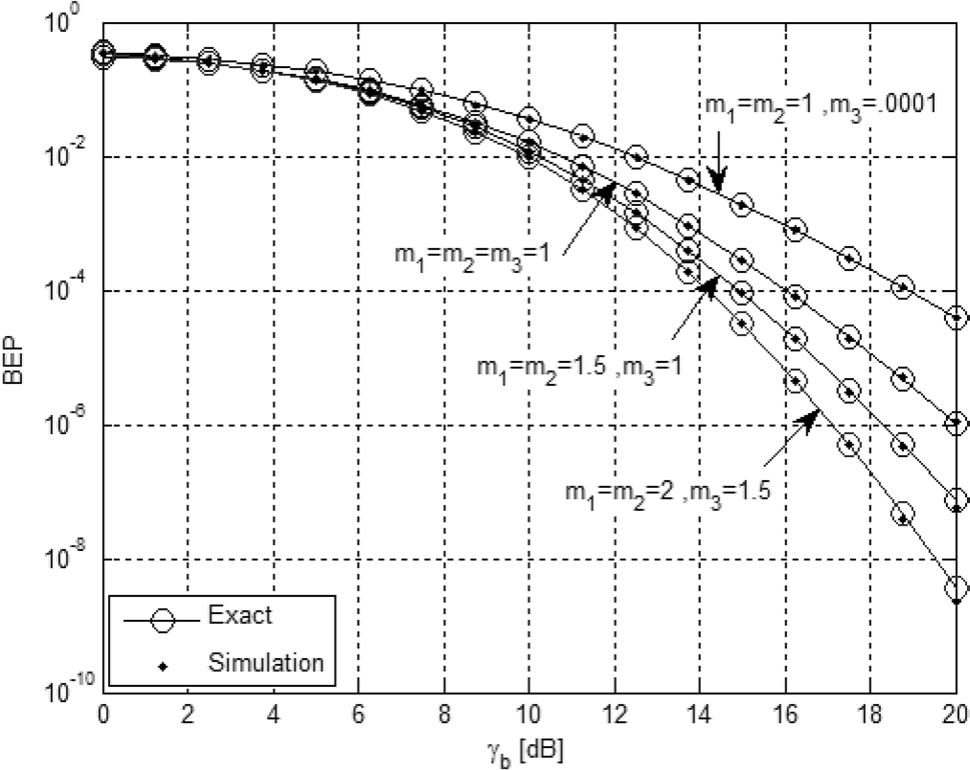
Impact of Nakagami-$m$ severity on the BEP performance of DCSK-CC with spreading factor $f = 64$.

Figures 3 and 4 depict the lower-bound BEP at the base station over Nakagami-$m$ fading channels for spreading factors $f = 32$ and $f = 64$, respectively. The close alignment between the numerically evaluated lower-bound curves and the simulation results supports the consistency of the adopted analytical treatment. In the revised discussion, the physically meaningful interpretation is restricted to the standard Nakagami-$m$ range, while the smallest shaping-parameter curve retained in the original figure is viewed only as a legacy stress-test case rather than as a standard propagation setting.

As shown in both figures, increasing the values of the fading parameter $m$ for the source-destination ($m_1$), relay-destination ($m_2$), and source-relay ($m_3$) links significantly enhances performance. This improvement is due to the fact that higher $m$ values represent less severe fading and better channel conditions, reducing the likelihood of deep fades and enhancing the reliability of signal detection.

In Fig. 4, for instance, at an SNR of 18 dB, the BEP for the uniform case $m_1 = m_2 = m_3 = 1$ is approximately $10^{-5}$. When the fading on the source-relay link improves to $m_3 = 1.5$, the BEP drops to nearly $10^{-7}$, demonstrating the crucial role of the relay link quality in cooperative systems. When the inter-user link becomes more severe, the cooperative gain decreases noticeably, highlighting the sensitivity of the considered scheme to relay-link reliability.

These results emphasize the importance of channel state variations in the three transmission links and validate that leveraging favorable fading conditions, especially at the relay, can significantly enhance the robustness of DCSK-based cooperative schemes.

**Fig. 5:**
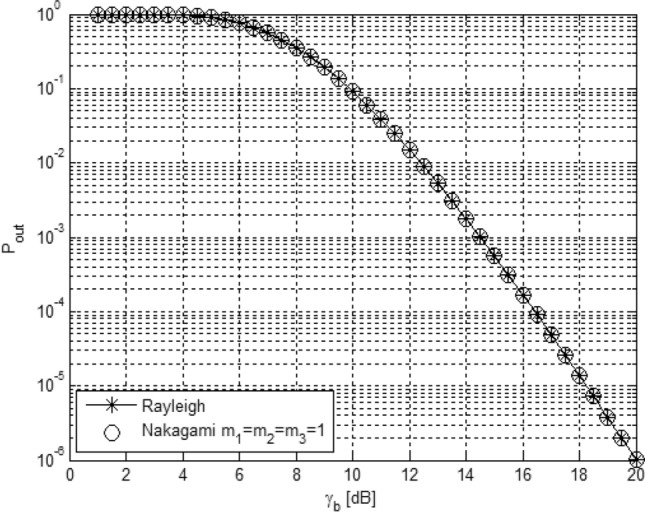
Outage performance of the DCSK-CC system over Rayleigh and Nakagami-$m$ fading channels for $m_1 = m_2 = m_3 = 1$.

**Fig. 6:**
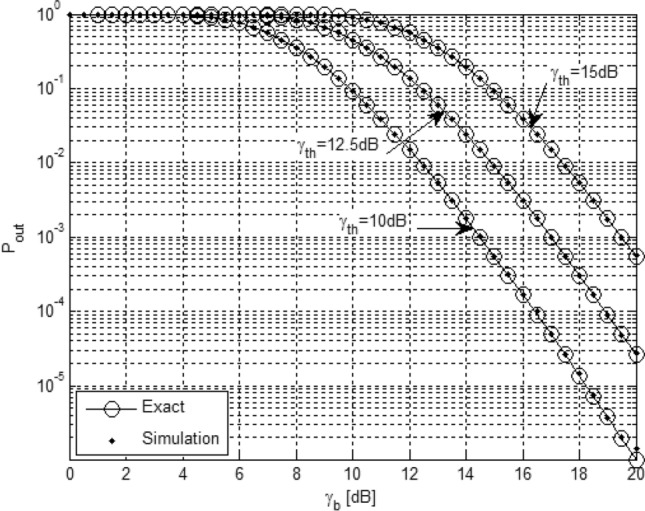
Outage performance of the DCSK-CC system under Rayleigh fading with threshold SNRs $\gamma _{\text {th}} = 10, 12.5,$ and $15$ dB.

**Fig. 7:**
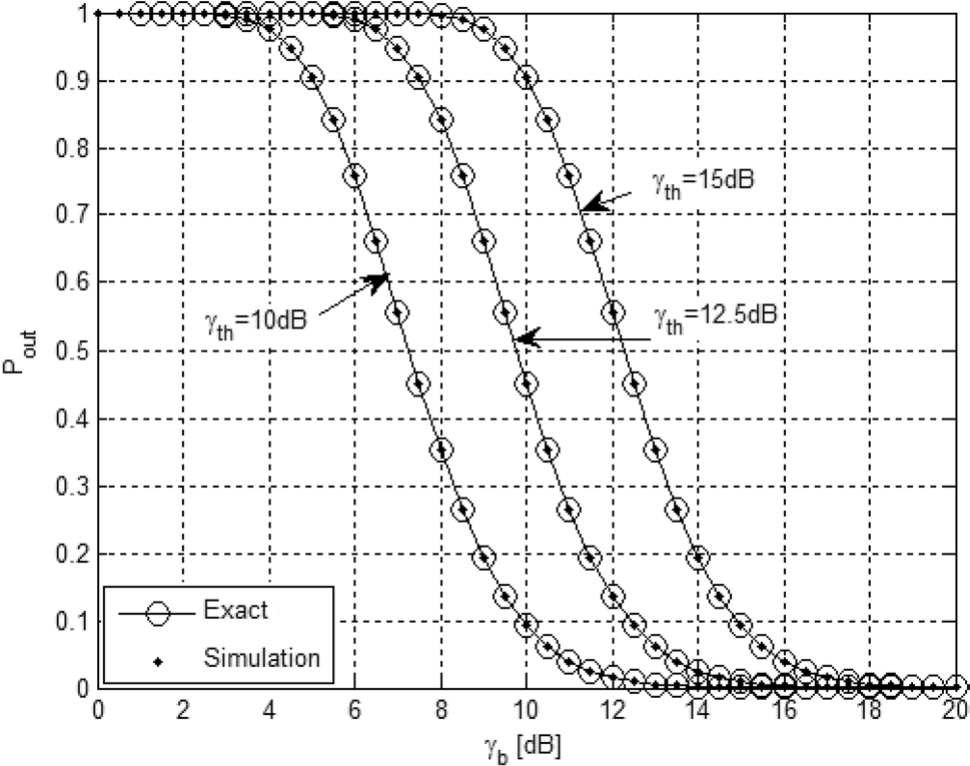
Outage performance of the DCSK-CC system under Nakagami-$m$ fading with threshold SNR values $\gamma _{\text {th}} = 10, 12.5,$ and $15$ dB.

**Fig. 8:**
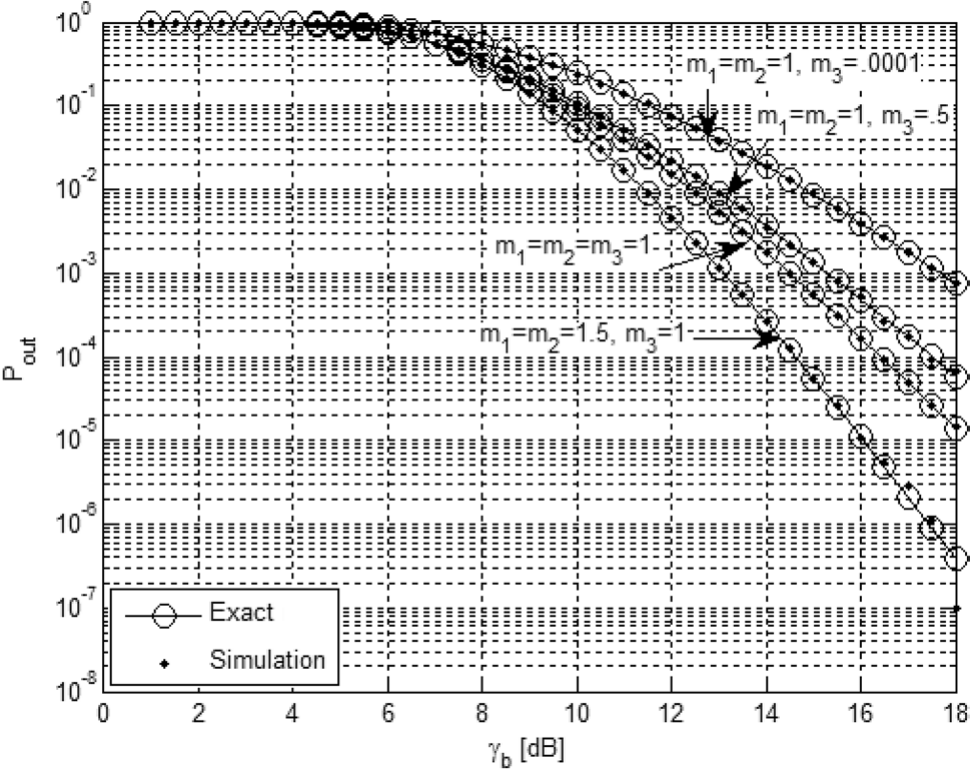
DCSK-CC system outage probability under Nakagami-$m$ fading with varying $m_1$, $m_2$, and $m_3$: Effect of link conditions at constant $\gamma _{\text {th}}$.

**Fig. 9:**
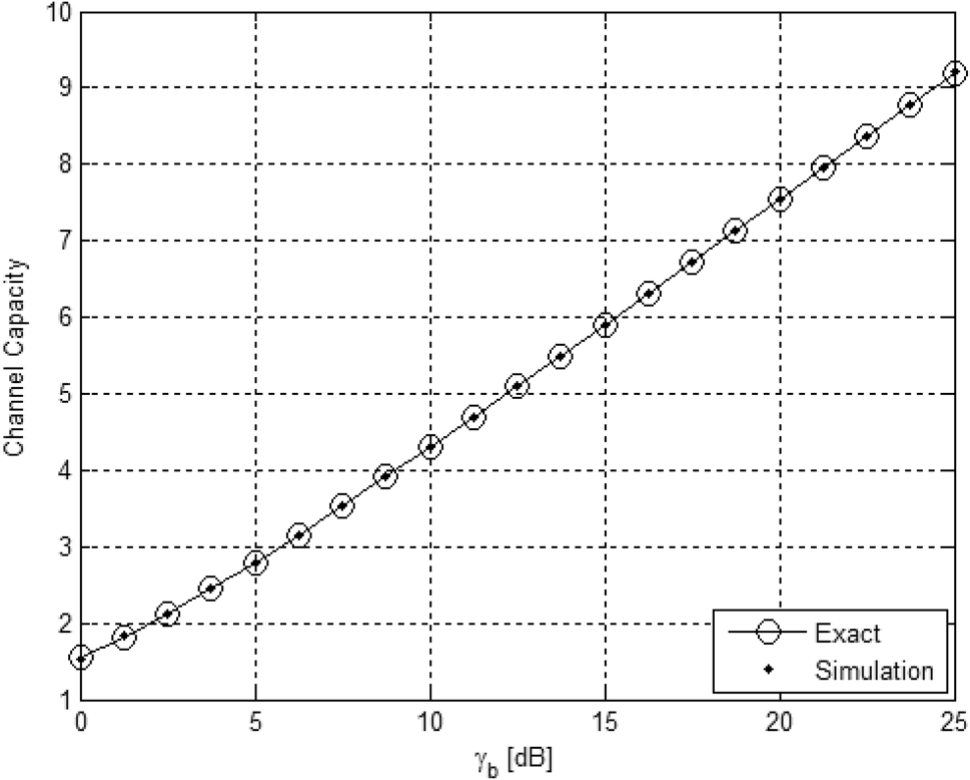
Average-capacity indicator of the DCSK-CC framework under Nakagami-$m$ fading with $m_1 = m_2 = 1$ and $m_3 = 1.5$: effect of improved relay-link conditions.

For reference, the non-cooperative DCSK counterpart corresponds to retaining only the odd-phase direct contribution, whereas the cooperative model combines odd- and even-phase observations through the equivalent SNR used in the present framework. Under ideal DF relaying, this additional contribution improves the received decision statistic and therefore provides the qualitative benchmark against which the cooperative behavior in Figs. 3, 4, 5, 6, 7, 8 and 9 should be interpreted. A dedicated quantitative benchmark plot against a non-cooperative or AF-relay alternative is left for future work.

Figure 5 illustrates the outage probability behavior of the proposed system model under Rayleigh fading and under Nakagami-$m$ fading with $m_1 = m_2 = m_3 = 1$. The nearly identical curves confirm that the adopted Nakagami-$m$ model reproduces the Rayleigh reference case when $m = 1$, thereby supporting the internal consistency of the evaluation framework. From a practical perspective, this result highlights that the Nakagami-$m$ model can seamlessly adapt to model both severe and moderate fading conditions, making it a versatile tool in analyzing real-world wireless environments. Moreover, it establishes a baseline for comparing the system’s behavior under varying severity levels of channel fading in the subsequent results.

Figure 6 presents the DCSK-CC system’s outage probability performance in a Rayleigh fading scenario for threshold SNR levels of 10, 12.5, and 15 dB. As expected, increasing the threshold leads to a rightward shift of the outage curve, indicating that higher SNR levels are required to avoid outage as the system’s reliability requirement becomes more stringent. This trend reflects a fundamental trade-off in system design: while raising $\gamma _{\text {th}}$ can support higher data rate or quality-of-service targets, it simultaneously increases the likelihood of outage in low-SNR environments. In addition, the close agreement between simulation and numerically evaluated reference results supports the consistency of the adopted outage evaluation.

Figure 7 shows the DCSK-CC outage probability over Nakagami-$m$ channels with varying threshold values $\gamma _{\text {th}} = 10, 12.5, 15$ dB. Similar to the Rayleigh case, increasing $\gamma _{\text {th}}$ shifts the outage curves to the right, indicating stricter reliability requirements. The rapid transition in the outage probability curves highlights how system performance responds to variations in the operating SNR when Nakagami fading is present. This behavior reflects the model’s adaptability to diverse wireless environments, where $\gamma _{\text {th}}$ may be tuned according to service-level agreements or energy constraints. In addition, the close alignment between the numerical reference curves and the simulations supports the robustness of the adopted evaluation framework under more general fading conditions.

Fig. 8 shows the outage probability of the DCSK-CC scheme over Nakagami- fading channels for various combinations of , , and , with a fixed threshold . The results clearly show that increasing the fading parameters leads to lower outage probabilities, as higher values correspond to less severe fading. Notably, the system is particularly sensitive to changes in , the parameter governing the source–relay link. When this link is heavily faded (e.g., ), system performance degrades substantially, underscoring the critical role of a reliable relay path. These findings suggest that enhancing the quality of the relay channel—through diversity schemes, positioning, or power allocation—can yield significant improvements in system reliability.        

Finally, Fig. 9 illustrates the channel capacity of the DCSK-CC scheme operating over Nakagami-$m$ fading channels with $m_1 = m_2 = 1$ and $m_3 = 1.5$, representing a scenario with improved fading conditions on the source–relay link. The numerical reference curve and the simulation results exhibit close agreement within the adopted equivalent-SNR model. As the SNR increases, the average-capacity indicator also increases, reflecting improved signal conditions at the destination. The result should therefore be interpreted as a comparative trend of the adopted indicator, rather than as a fully normalized spectral-efficiency claim for the complete protocol.

## Conclusion and future work

This paper investigated a two-user differential chaos shift keying cooperative communication framework with decode-and-forward relaying over Rayleigh and Nakagami-*m* fading channels. Within the adopted model, outage probability and an average-capacity indicator were evaluated from the equivalent received-SNR formulation, while the bit error probability at the base station was treated through a lower-bound analytical characterization supported by Monte Carlo simulation. The results consistently showed that milder fading conditions improve reliability and that the inter-user link quality has a direct influence on the cooperative gain.

The revised manuscript intentionally limits its conclusions to the analytical setting studied here. In particular, the reported capacity quantity should be interpreted as a comparative indicator based on the equivalent SNR, not as a fully normalized spectral-efficiency expression for the full DCSK-CC protocol. Likewise, the BEP result at the base station is a lower-bound treatment obtained under idealized relay-decoding assumptions.

Overall, the analytical and simulation trends were found to be consistent within the adopted assumptions. Future work should extend the study by including explicit benchmark comparisons with non-cooperative DCSK and alternative relaying strategies, by incorporating synchronization and implementation losses, and by examining broader fading models and protocol-normalized throughput measures.

## Data Availability

No datasets were generated or analyzed during the current study.

## Appendix A: Adopted equivalent-SNR PDF representation

For the Nakagami-$m$ case, the path-level SNR is modeled by the Gamma distribution

25 $$\begin{aligned} f(\gamma _l)=\frac{m_l^{m_l}\gamma _l^{m_l-1}}{\Gamma (m_l)\bar{\gamma }_l^{m_l}} \exp \!\left( -\frac{m_l\gamma _l}{\bar{\gamma }_l}\right) . \end{aligned}$$

After combining the link contributions under the adopted equivalent-SNR model, the resulting base-station PDF is written compactly as

26 $$\begin{aligned} f_{\gamma _b^{\textrm{BS}}}(\gamma )= c_{\textrm{BS}}e^{-b_1\gamma }\gamma ^{a_{\textrm{BS}}-1} {}_1F_1\!\left( b_{\textrm{BS}},a_{\textrm{BS}},\beta _{\textrm{BS}}\gamma \right) . \end{aligned}$$

This compact notation is used in the main text to keep the outage and average-capacity expressions concise and to avoid ambiguity between path-level and link-level parameters.

## Appendix B: Lower-bound BEP evaluation

The conditional BEP adopted in the manuscript is

27 $$\begin{aligned} P_e(\gamma _b)=\frac{1}{2}\,\textrm{erfc}\!\left[ \left( \frac{4}{\gamma _b}+\frac{2\beta }{\gamma _b^2}\right) ^{-1/2}\right] . \end{aligned}$$

At the base station, a lower-bound treatment is obtained by assuming ideal inter-user decoding and symmetric weighted-energy contributions from the odd and even phases. Under these assumptions, the average BEP is evaluated numerically as

28 $$\begin{aligned} \bar{P}_e = \int _0^\infty P_e(\gamma )\,f_{\gamma _b^{\textrm{BS}}}(\gamma )\,d\gamma . \end{aligned}$$

This is the quantity used to generate the lower-bound reference curves discussed in the results section.

## Appendix C: Outage-probability evaluation

Using the adopted equivalent-SNR PDF in Eq. (26), the outage probability is evaluated from

29 $$\begin{aligned} P_{\textrm{out}}(\gamma _{\textrm{th}})=\int _0^{\gamma _{\textrm{th}}} f_{\gamma _b^{\textrm{BS}}}(\gamma )\,d\gamma . \end{aligned}$$

For the Rayleigh case, the same expression is evaluated after setting the corresponding Nakagami-$m$ parameters to unity. The numerical reference curves in the manuscript are obtained directly from this integral representation.

## Appendix D: Average-capacity indicator evaluation

The average-capacity indicator used in the manuscript is computed as

30 $$\begin{aligned} \bar{C}=\textrm{BW}\int _0^\infty \log _2(1+\gamma )\,f_{\gamma _b^{\textrm{BS}}}(\gamma )\,d\gamma . \end{aligned}$$

The Rayleigh reference case is again obtained by setting $m_1=m_2=m_3=1$. The reported curves are based on direct numerical evaluation of the above integral and are interpreted comparatively within the equivalent-SNR framework adopted in the paper.
